# Non-volatile organic compounds in exhaled breath particles correspond to active tuberculosis

**DOI:** 10.1038/s41598-022-12018-6

**Published:** 2022-05-13

**Authors:** Dapeng Chen, Noella A. Bryden, Wayne A. Bryden, Michael McLoughlin, Dexter Smith, Alese P. Devin, Emily R. Caton, Caroline R. Haddaway, Michele Tameris, Thomas J. Scriba, Mark Hatherill, Sophia Gessner, Digby F. Warner, Robin Wood

**Affiliations:** 1 Zeteo Tech, Inc., Sykesville, MD USA; 2 South African Tuberculosis Vaccine Initiative, Institute of Infectious Disease and Molecular Medicine and Division of Immunology, Department of Pathology, University of Cape Town, Cape Town, South Africa; 3 SAMRC/NHLS/UCT Molecular Mycobacteriology Research Unit, Institute of Infectious Disease and Molecular Medicine and Division of Medical Microbiology, Department of Pathology, University of Cape Town, Cape Town, South Africa; 4 Desmond Tutu HIV Centre, Institute of Infectious Diseases and Molecular Medicine, University of Cape Town, Cape Town, South Africa

**Keywords:** Biochemistry, Biomarkers, Medical research

## Abstract

Human breath contains trace amounts of non-volatile organic compounds (NOCs) which might provide non-invasive methods for evaluating individual health. In previous work, we demonstrated that lipids detected in exhaled breath aerosol (EBA) could be used as markers of active tuberculosis (TB). Here, we advanced our analytical platform for characterizing small metabolites and lipids in EBA samples collected from participants enrolled in clinical trials designed to identify molecular signatures of active TB. EBA samples from 26 participants with active TB and 73 healthy participants were processed using a dual-phase extraction method, and metabolites and lipids were identified via mass spectrometry database matching. In total, 13 metabolite and 9 lipid markers were identified with statistically different optimized relative standard deviation values between individuals diagnosed with active TB and the healthy controls. Importantly, EBA lipid profiles can be used to separate the two sample types, indicating the diagnostic potential of the identified molecules. A feature ranking algorithm reduced this number to 10 molecules, with the membrane glycerophospholipid, phosphatidylinositol 24:4, emerging as the top driver of segregation between the two groups. These results support the use of this approach to identify consistent NOC signatures from EBA samples in active TB cases. This suggests the potential to apply this method to other human diseases which alter respiratory NOC release.

## Introduction

Human exhaled air contains water vapor and trace amounts of organic materials including volatile organic compounds (VOCs), non-volatile organic compounds (NOCs), and particulate matter including microbes^1–5^. Molecular characterization of these organic compounds provides a noninvasive method for the investigation of human diseases^5^. Typically, human breath analysis uses a pre-concentration step to account for the low amounts of organic and biological molecules, usually in the parts per billion to parts per trillion range^1^.

Since small gas molecules can be identified by relatively simple MS-based platforms, human breath analysis has focused on VOCs^6^. For example, an MS with moderate resolution (R = 1000) has the capacity to resolve the molecular patterns of gas molecules. However, VOC analysis has a fundamental drawback as the endogenous metabolic origins of the molecules detected are poorly understood^6^. Consequently, changes in VOC patterns might be caused by factors including internal gut and airway bacteria, host physiological state, or exogenous influences such as drinking, smoking, and eating^7^. Changes in VOCs can also be caused by the inhalation of gases generated from environmental sources such as breath samplers and bioaerosols^6,7^. As a result, changes in VOC fingerprints acquired from non-targeted sensors, such as MS and ion mobility spectrometry, do not necessarily represent individual phenotypes and could be falsely interpreted as disease biomarkers by pattern recognition tools, limiting their application in medical diagnostics^6,7^. Therefore, there is a need for a comprehensive assessment of biomolecules in human breath for diagnostics, individual phenotyping, and respiratory disease monitoring.

NOCs in human exhaled air include metabolites, lipids, and proteins contained in exhaled water vapor (exhaled breath condensate, EBC) and exhaled breath aerosols (EBA)^5^. To date, the major setbacks to achieving a better understanding of nonvolatile molecules have been instrument limitations and technical difficulties in the analysis of these large molecules. However, the introduction of high-frequency and ultrahigh-resolution MS has overcome these hurdles, creating exciting possibilities for the characterization of NOCs in human breath^8^. Increase evidence suggests that particles contained in human exhaled air are associated with disease transmission, contributing to public health risks such as seasonal influenza, tuberculosis (TB), and the COVID-19 pandemic^4,9^. Given these findings, collection and characterization of NOCs are critical to establishing potential biomarkers for the detection of respiratory infection, medical diagnosis, disease screening, and performance metrics^5,10–12^. In previous work, we demonstrated that detecting lipid markers using high-resolution MS could be used to identify active TB cases^5^. Here, we applied advanced analytical approaches to 200 EBA samples acquired from clinical trials and evaluated the correlation between respiratory NOCs and TB status^13^.

## Results

### NOC analysis of EBA samples from CORTIS participants

**Figure 1:**
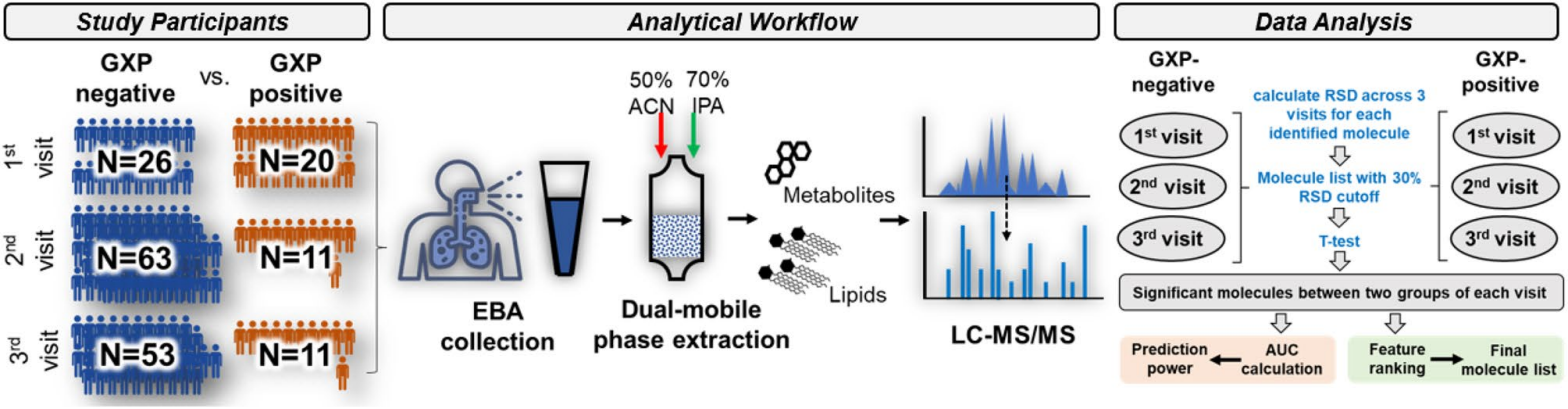
CORTIS participant information and workflow for identification of NOCs from EBA samples. The total number of CORTIS study participants who tested negative (GXP-negative) or positive (GXP-positive) with GeneXpert MTB/RIF are shown. EBA samples were collected as previously described^5^. A dual-mobile phase extraction method, utilizing 50% ACN and then 70% IPA, enabled extraction of small metabolites and lipids from the EBA samples. Extracted molecules were processed with LC–MS/MS and identified via database matching. Relative standard deviation (RSD) of each identified molecule was calculated for each group. Molecules with < 30% RSD were used for *t*-test between GXP negative and positive of each visit. Molecules showing statistical differences in all visits were used to generate receiver operating characteristic (ROC) curve and area under the curve (AUC) calculation. A feature ranking algorithm was used to show the importance of identified molecules.

In total, 99 CORTIS trial participants, classified as either GeneXpert (GXP)-negative or GXP-positive, were included in this study. 200 EBA samples (including revisits for some participants) were collected at different clinical visits (Table 1, Fig. 1, and Supplementary Table 1). Age, gender, and smoking status did not significantly correlate with TB status. All the study subjects were HIV negative. On the 1st visit, there were 26 GXP-negative participants and 20 GXP-positive participants; on the 2nd visit, there were 63 GXP-negative participants and 11 GXP-positive participants; and on the 3rd visit, there were 53 GXP-negative and 11 GXP-positive participants. All EBA samples were processed through 0.2 µm membrane filtration (see methods) in which the bioaerosol pellet was separated from the supernatant. The concept of using dual-mobile phase extraction was based on the hydrophobicity of molecules. Since lipids are non-polar molecules, 70% isopropyl alcohol (IPA) is used for elution^14^. The dual-mobile phase method enables us to extract both small metabolites (using 50% acetonitrile (ACN) solvent) and lipids (using 70% IPA solvent) from the same EBA sample, deepening the molecular identification and analysis in LC–MS/MS (Fig. 1).

**Table 1:** Study participant information.

TB status	Subjects	Gender		Age	HIV status		Smoking status	
					Positive	Negative	Smoker	Non-smoker
Negative	73	Female	47 (64%)	29.1 ± 10.3	0	45	16	29
		Male	26 (36%)		0	26	22	4
Positive	26	Female	7 (27%)	33.7 ± 10.6	0	7	2	5
		Male	19 (73%)		0	19	15	4
	*p* value			0.92	NA		0.89

### Validation of the dual-mobile phase extraction method

**Figure 2:**
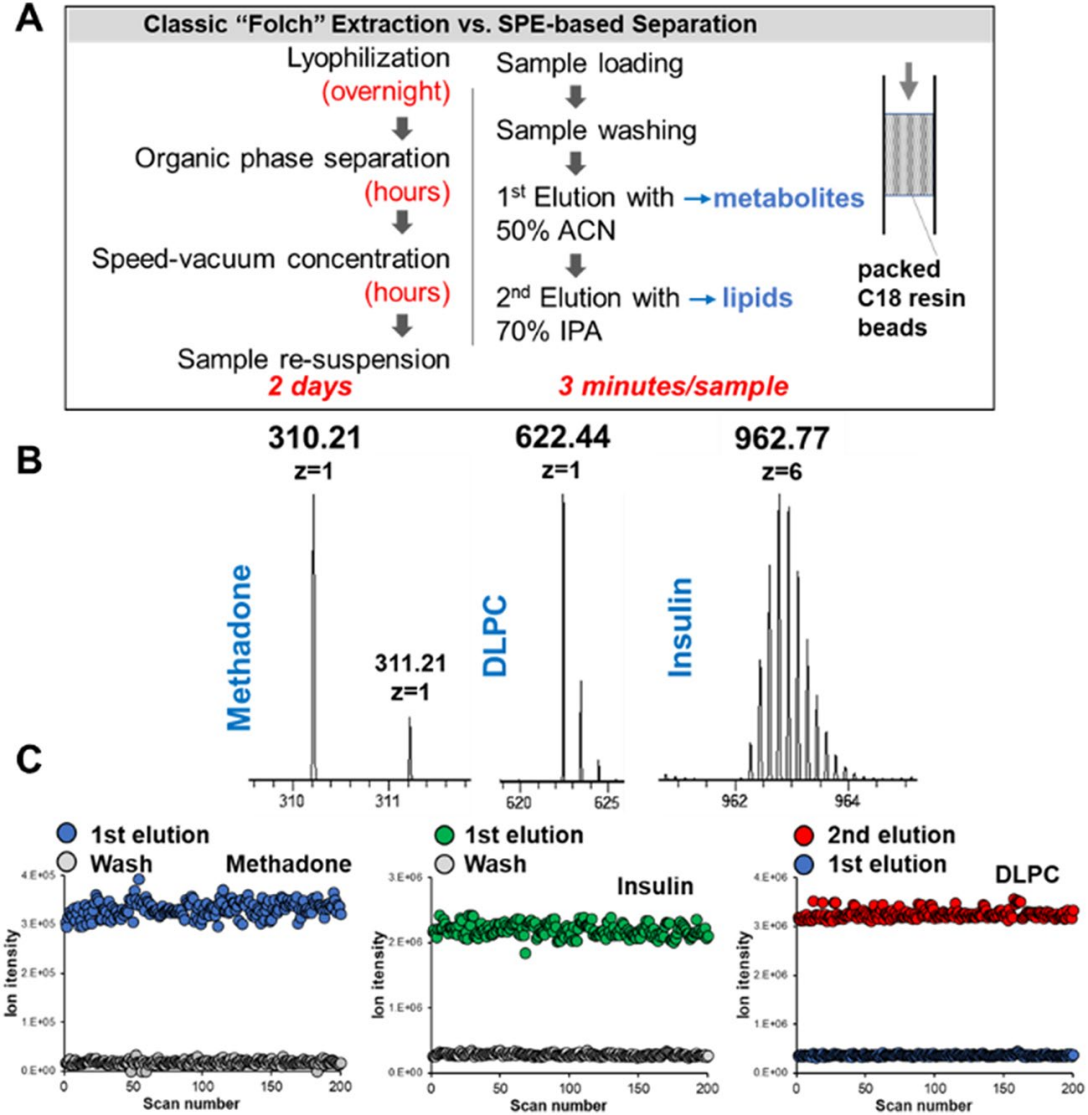
Method development for the separation of small molecules and lipids from one sample using duel mobile phase solvents. (**A**) Comparison between the classic Folch method and the solid-phase extraction (SPE)-based method developed in this work, which incorporates dual-mobile phase extraction. (**B**) Mass spectra of three representative molecules were used to validate the dual-mobile phase extraction method. (**C**) Recorded ion intensities of three representative molecules in either washing or eluting solutions.

In our previous work, lipids were extracted using a classic Folch solvent separation method^5^. Since the EBA samples were collected in ~ 10 mL of buffer solution, they required overnight lyophilization and centrifugation for extraction (Fig. 2A). These steps are resource-intensive and difficult to manage at scale; therefore, in the current study, we applied our newly developed solid-phase extraction (SPE) approach, which uses a C18 resin column as the capture matrix. For the sample preparation, EBA samples were loaded directly into the column. After washing, small metabolite molecules and proteins, which are polar, were eluted using ACN (1st elution). Lipids, which are nonpolar, were eluted with 2-propyl alcohol (2nd elution, Fig. 2A). Three representative molecules—methadone, dilauroyl-sn-glycero-3-phosphorylcholine (DLPC), and insulin—were used for method validation (Fig. 2B). Methadone and insulin were only detected in the 1st elution sample, not in the washed sample (Fig. 2C, red and green dots). DLPC signals were detected in the 2nd elution sample, which suggests efficient capture of lipid molecules by the C18 resin. The SPE-based sample preparation was rapid, with the separation process taking ~ 3 min *per* sample, and the use of a C18 resin column makes it adaptable to automation. Notably, dual mobile phases for polar and nonpolar molecule separation have been used by others, reinforcing the reliability and reproducibility of this approach^15^.

### Molecular profiles of EBA samples with liquid chromatogram and MS

**Figure 3:**
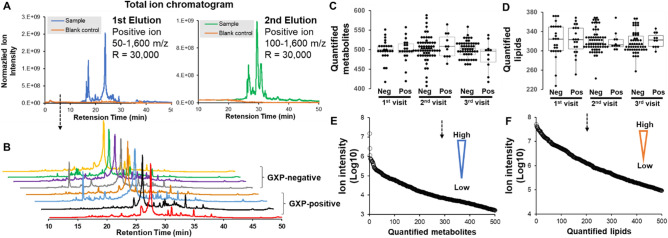
Overview of molecular profiles of EBA samples with liquid chromatography and high-resolution MS. (**A**) Total ion chromatograms of control samples and EBA samples. (**B**) Representative total ion chromatograms of small metabolites from GXP-positive and GXP-negative participants. (**C**) Feature distributions of small metabolites from three visits of GXP-positive and GXP-negative participants. (**D**) Feature distributions of lipids in three visits of GXP-positive and GXP-negative participants. (**E**) Dynamic range of small metabolites detected across all study participants. (**F**) Dynamic range of lipids detected across all study participants.

The total ion chromatogram of extracted small metabolites and lipids in EBA samples showed that molecular signals were acquired in EBA samples when compared to blank samples (Fig. 3A, blue, green, and orange lines). Representative total ion chromatograms of small metabolites in EBA samples from GXP-positive and GXP-negative participants revealed no obvious differences between the two groups, suggesting a deeper analysis was required (Fig. 3B). In total, ~ 500 features were extracted from small metabolite analysis and ~ 330 features were extracted from lipid analysis (Fig. 3C,D). No statistical difference was observed between the two groups based on the feature numbers. Both small metabolite and lipid analyses with MS achieved outstanding dynamic range: ~ 5 magnitudes in small metabolite profiles and ~ 4 magnitudes in lipid profiles (Fig. 3E,F). The high similarity among LC–MS profiles and the identified molecule numbers suggested decent technical repeatability of our method.

### Selection of definitive molecules in each study group

**Figure 4:**
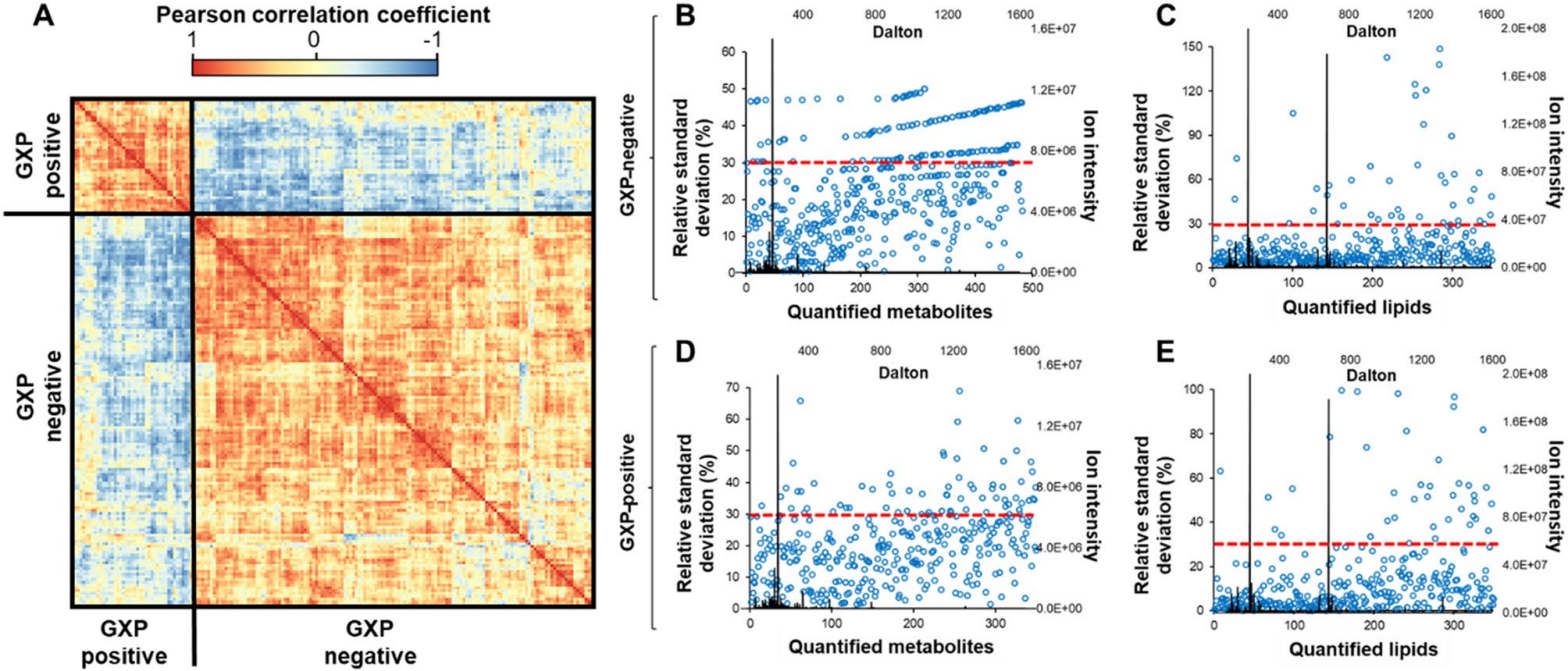
Overview of study participant correlation and individual feature deviations. (**A**) Global correlation heatmap of study participants. Distribution of relative standard deviation values of metabolites and lipids in (**B**, **C**) GXP-negative and (**D**, **E**) GXP-positive study participants. The red dashed lines indicate 30% relative standard deviation threshold.

**Figure 5:**
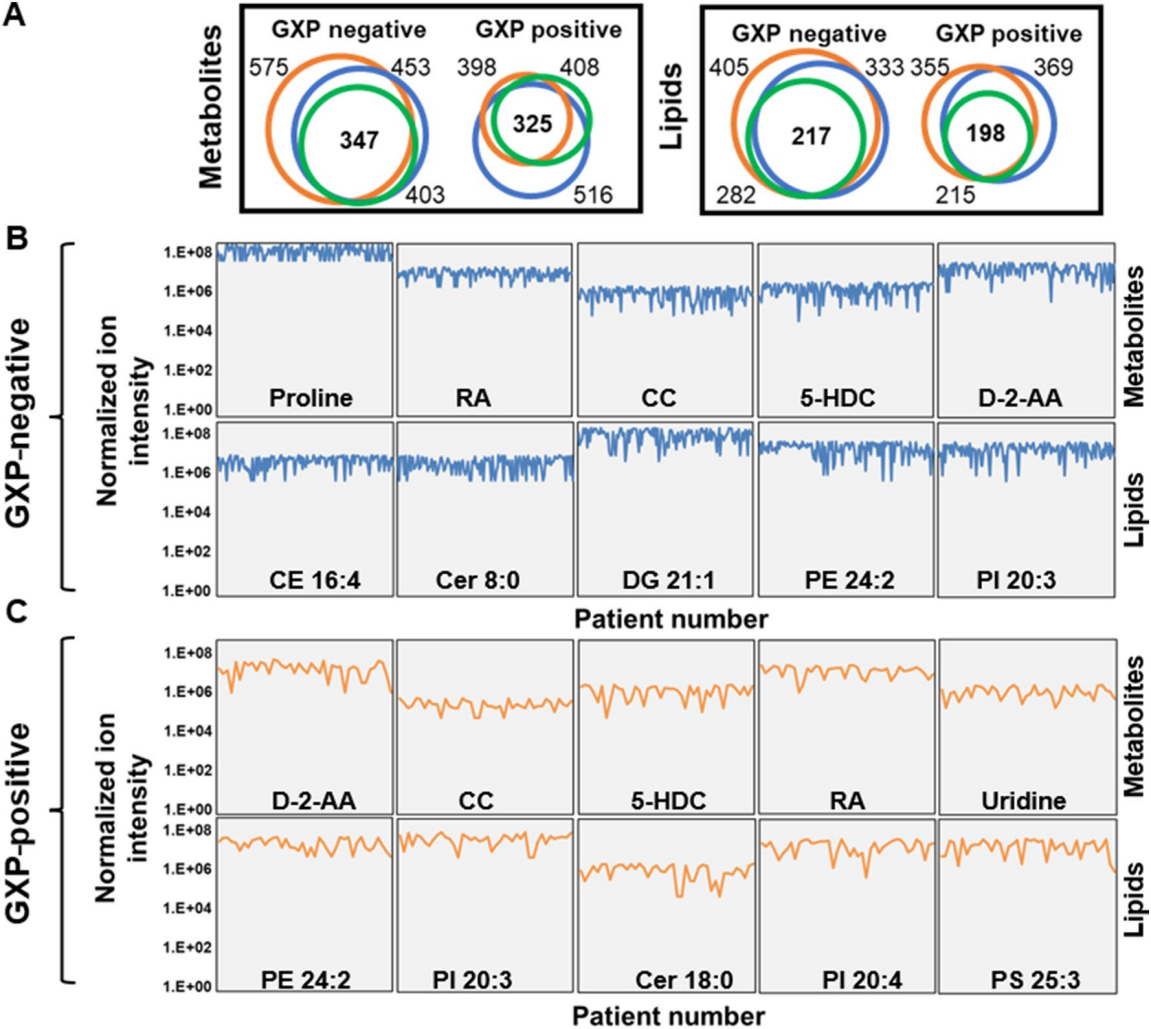
Identification of definitive features in GXP-negative and GXP-positive groups. (**A**) Numbers of metabolites and lipids identified in each study group at each visit. (**B**) The five most abundant metabolites and lipids identified in (**B**) GXP-negative and (**C**) GXP-positive study participants. Blue, green, and orange circles indicate the numbers of identified molecules at each visit.

A global correlation heat map demonstrates that molecular profiles could be used to segregate the two groups of study participants with GXP-negative and GXP-positive participants exhibiting clear correlations by Pearson correlation coefficient analysis (Fig. 4A). The relative standard deviation of each feature of either GXP-negative or GXP-positive samples was calculated (Fig. 4B–E). A 30% threshold was used to select features in each group for statistical analysis (Fig. 4B–E, red line), resulting in 347 small metabolites and 217 lipids in the GXP-negative group, and 325 small metabolites and 198 lipids in the GXP-positive group (Fig. 5A). The distributions of ion intensity for 10 representative molecules in each participant group were identified as proline, all-trans-retinoic acid (RA), chalcone (CC), 5-hydroxyindole-3-acetic acid (HIAA), D-2-Aminobutyric acid (D2AA), uridine, cholesteryl ester (CE) 16:4, ceramide (Cer) 8:0, diacylglycerol (DG) 21:1, phosphatidylethanolamine (PE) 24:2, phosphatidylinositol (PI) 20:3, and phosphatidylserine (PS) 5:3 (Fig. 5 B,C).

### Identification of 22 metabolite and lipid molecules associated with active TB status

**Figure 6:**
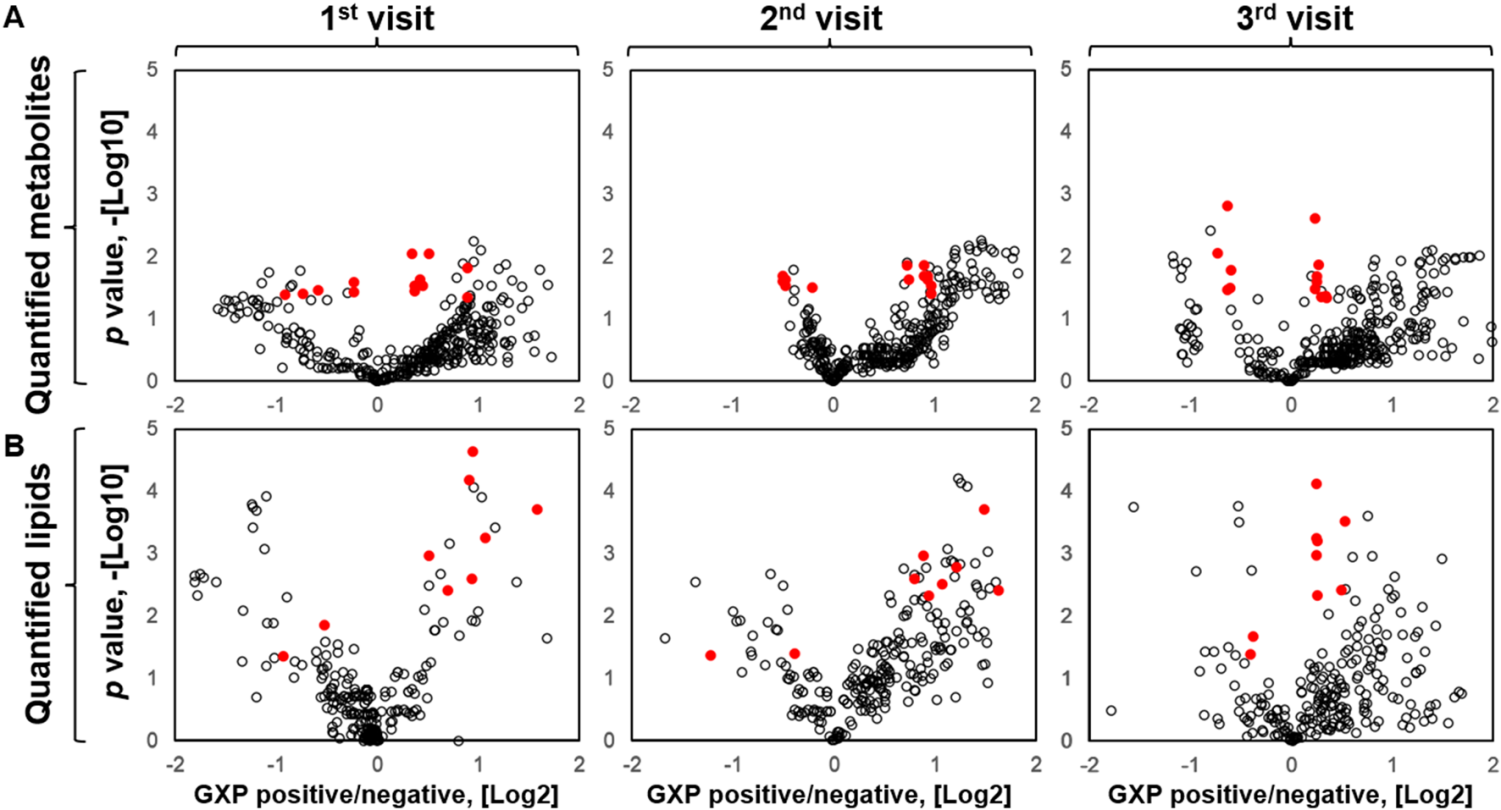
Statistical differences in molecular profiles for GXP-negative and GXP-positive groups at each visit. Volcano plots of GXP-positive/GXP-negative fold change and *t*-test *p* values for (**A**) metabolites and (**B**) lipids at each visit. Red dots indicate that the identified molecules were statistically different between the two groups in all three visits. The blue dashed lines indicate *p-*value of 0.05, which is log-transferred in the y-axis: − Log_10_(0.05) = 1.3.

**Figure 7:**
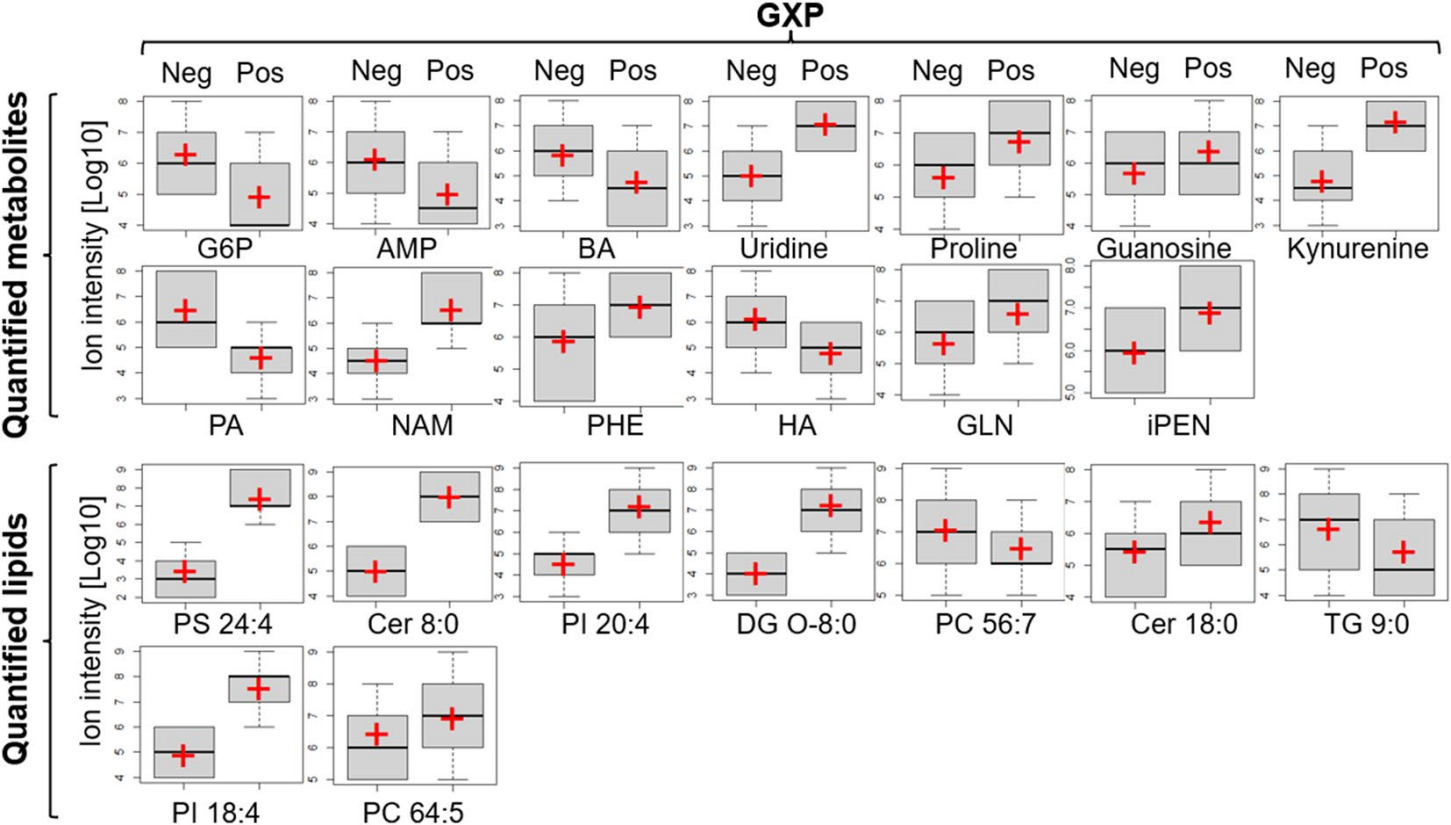
Standard error distribution of metabolites and lipids that were significantly different in GXP-negative and GXP-positive groups across all three visits. Red crosses indicate the mean values. G6P: d-glucosamine-6-phosphate; AMP: adenosine monophosphate; BA: butanoic acid; PA: palmitic acid; NAM: n-acetylmuramic acid; PHE: phenylalanine; HA: hyocholic acid; GLN: glutamine; iPEN: isopentenyladenine; PS: phosphatidylserine; Cer: ceramides; PI: phosphatidylinositol; DG: diacylglycerol; PC: phosphatidylcholine; TG: triglycerides.

Metabolites and lipids that were statistically significant by *t*-test are identified in the volcano plots (Fig. 6A,B, Supplemental Table 2). The molecules that were statistically significant at all three visits are marked red dots (Fig. 6A,B). In summary, 22 molecules—including 13 metabolites and 9 lipids—were identified that were statistically significant in 2-tailed *t*-test between the two groups at all three visits (Figs. 6A,B and 7). The data normal distribution of 22 molecules was confirmed with Shapiro–Wilk test (Supplemental Table 3). The threshold in *p* values for selecting these 22 molecules in the volcano plots was defined as *p* < 0.05 (− Log10 = 1.3). We did not define the threshold for the fold changes. If the threshold is defined as fold change larger than ± 1.5 (log2 = 0.58), fewer molecules will meet this criterion (Supplemental Table 2).

**Figure 8:**
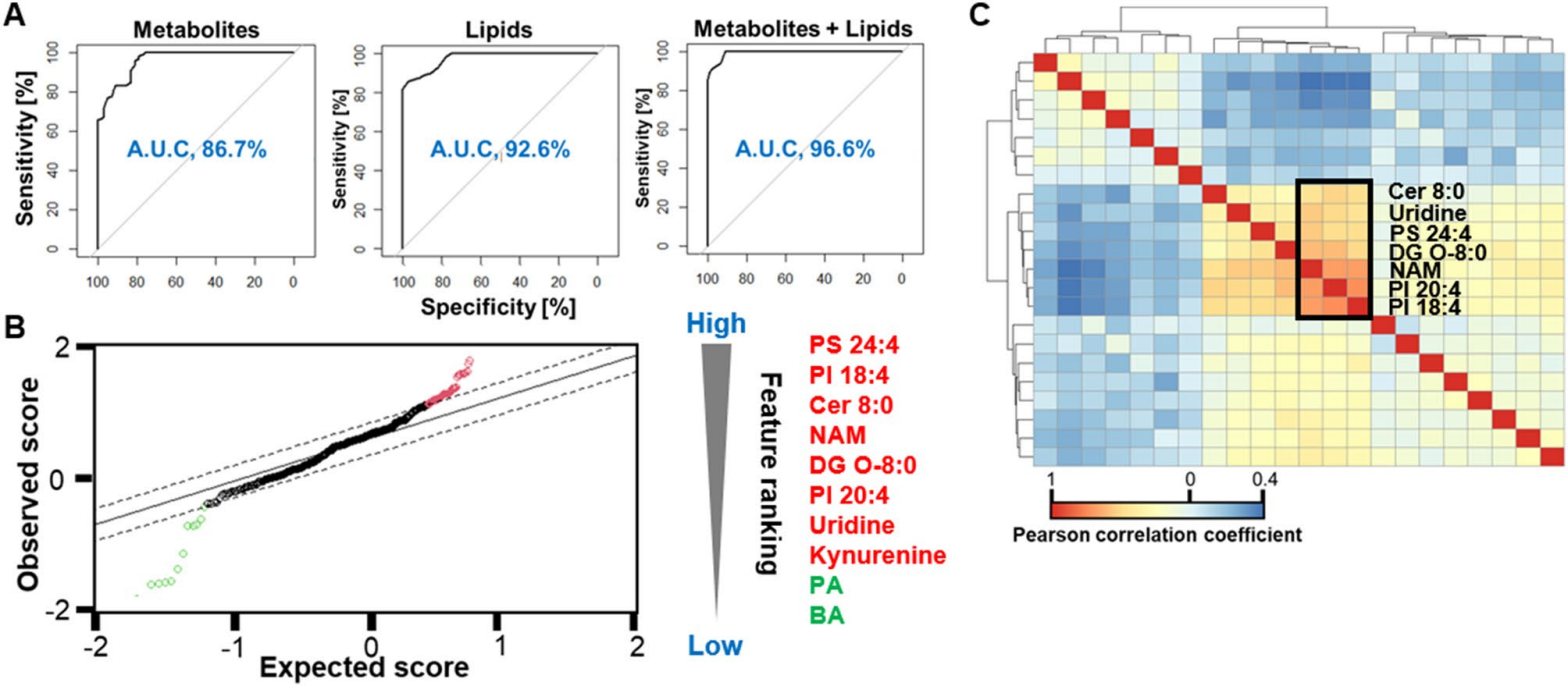
Significance analysis of metabolites and lipids differentiating GXP-negative and GXP-positive groups. (**A**) AUC values for logistic regression models using identified molecules. (**B**) Feature ranking analysis of metabolites and lipids based on significance analysis of microarrays (SAM) algorithm. (**C**) Correlation heatmap of metabolites and lipids based on Pearson correlation coefficient analysis. Red dots indicate increasing and green dots indicate decreasing in GXP-positive.

We then evaluated the utility of the identified metabolites and/or lipids to distinguish GXP-negative from GXP-positive participants by generating ROC curves and calculating AUC values (Fig. 8A). The AUC for metabolites was ~ 87% (95% confidence interval: 0.832–0.919) and 93% (95% confidence interval: 0.889–0.971) for lipids to differentiate between GXP-negative and GXP-positive study participants. When combined with metabolites and lipids, the AUC was slightly higher than using lipids only, ~ 97% (95% confidence interval: 0.926–0.986) for the differentiating between GXP-negative and GXP-positive participants (Fig. 8A). The correlation between the identified molecules was investigated by Pearson correlation coefficient analysis (Fig. 8C), with the heatmap identifying seven molecules (Cer 8:0, uridine, PS 24:4, DG O-8:0, NAM, PI 20:4, PI 18:4) with the highest correlation. In our previous work, we used significance analysis of microarrays (SAM) to identify those features with the most separation power^5^. Applying SAM analysis to the current dataset revealed 10 molecules (Fig. 8B) that were significantly different between GXP-positive and GXP-negative samples, with PS 24:4 emerging as the top driver of segregation between the two groups.

## Discussion

Investigations of the molecular composition of human breath have predominantly focused on VOCs^6^. This bias is driven largely by the availability of analytical tools to extract and evaluate VOCs in a comprehensive manner, such as gas absorption tubes, electronic chemical sensors, and low-resolution MS^6,7^. As a result, thousands of gas molecular signatures can be acquired and used for pattern recognition and fingerprint analysis^1^. However, there is a critical need for improved tools for NOC analysis given their biological relevance, including both host and pathogen proteins, lipids, metabolites, and nucleic acids^2–5,16,17^. In this study, we advanced the analytical approach to extract NOCs from EBA samples that were collected from well-defined study groups^13^. Coupling our dual mobile phase extraction methodology with high-resolution MS, rich molecular signatures were acquired. This methodology allows for identification of individual molecules providing high separation power. We demonstrate here the potential use of metabolite and lipid profiles to differentiate active TB cases from other study subjects.

The presence of polar metabolites and non-polar lipids in NOCs poses a challenge in applying a universal extraction method for the comprehensive characterization of molecules in EBA. Phase extraction methods based on organic solvents, such as Folch extraction, can be used, but are resource-intensive and time-consuming as several organic solvents and drying procedures are required^5^. While improvements have been made, solvent-based molecule extraction methods are not useful for sample processing at scale. Inspired by absorption materials used in conventional liquid chromatography and solid-phase extraction, we developed a dual-mobile phase extraction method, using a self-packed C18 column to separately capture both polar and non-polar molecules from the same sample^14^. The method is based on the different affinity of metabolites and lipids to the C18 functional group. Conventional acetonitrile can be used to elute metabolites, while the harsher solvent, IPA, is required to elute non-polar lipids^14^. In fact, switching the solvent system to achieve a more comprehensive analysis has been done previously in liquid chromatography^15^. We validated our approach using three different representative molecules: methadone for metabolites; DLPC, a lung surfactant, for lipids; and insulin for small peptides and proteins. Our results indicate that the dual-phase extraction method is rapid and with high recovery rate. Using this approach, we extracted hundreds of molecular features from individual EBA samples. Although proteins are of great interest for biological analysis given that their origin (host *versus* pathogen) can be easily ascribed, our initial analysis yielded minimal to no protein content in EBA samples. Consequently, our extraction and analysis focused on metabolites and lipids—though it is likely that an additional filter cutoff may be applied to separate small metabolites and large protein molecules.

MS generates thousands of signals suggesting feature pre-selection is required to achieve a confident analysis. In our study, we aimed to select the molecules most definitively associated with disease status by identifying precise and repeatable individual molecules in GXP-negative and GXP-positive study groups. To start, background ion signals and signals contributing to non-biological information were first excluded by the control blank samples. In addition, to improve data repeatability, a desired relative standard deviation cutoff was applied to identified molecules in each group, resulting in a list of features that showed the least variation. The advantage of this approach is that molecules of statistical significance will be more reproducible as more study subjects are available. However, the main drawback for this feature-reducing approach, especially in studies with small subject numbers, is the potential to eliminate important features^5^. However, the limitations of this approach should be recognize. The main drawback is the possibility of eliminating important features, a real risk in studies with small numbers of subjects^5^. An alternative solution is to assign the false-discovery rate (FDR) to each feature and include only significant features in data analysis.

TB molecular diagnosis relies heavily on nucleic acid amplification technologies (NAATs) such as GeneXpert MTB/RIF assay^18^. An acknowledged limitation of this assay is its dependence on the production of high-quality sputum specimen^18^. Therefore, there is interest in the development of a non-sputum specimen collection method and molecular diagnositic assay that does not require expensive reagents^5^. Considering MS has been used in the clinical labs for microorganism identification, we evaluated molecular signatures revealed by MS acquired in EBA samples for potential biomarkers in active TB cases.

Our approach identified 22 molecules that were statistically different between GXP-negative and GXP-positive cases. For biomarker discovery using metabolomics and lipidomics, it is expected that a prediction model can be developed that groups molecules to predict a diagnosis. However, to avoid over-fitting, the prediction model needs to include the simplest combination of features that provide the most precise prediction. Several artificial intelligence/machine learning (AI/ML) programs, such as neural networks and decision trees, can be used for this purpose^5,19^. Nevertheless, in this study we investigated the correlation of metabolites and lipids and the importance of each molecule via Pearson’s and SAM. The results indicated lipid molecules offered better separation power than metabolites. More specifically, lipid molecules showed a stronger correlation as well as ranked higher in the feature importance evaluation. This notion is further supported by the ROC curves and AUC calculation as lipids alone contributed to ~ 93% segregation with only a slight improvement in the score with the addition of metabolite markers. Our results need to be carefully interpreted for diagnostic applications. More analysis, such as multiple comparison correlations, including training/validating sets, evaluating over/under-fitting, and sample size estimation, should be added to address the potential clinical implications.

We previously demonstrated that molecules identified from human exhaled air samples overlap with molecules present in blood. In the current work, we identified molecules which have been reported in other studies that used blood specimens for biomarker discovery in active TB^20–22^. For example, Feng and coauthors investigated serum metabolic profiles of 271 participants using LC–MS and reported that 12 metabolites contributed to the segregation between active TB and control groups with multiple logistic regression analysis^21^. Among those metabolites, palmitic acid was found to be lower in the active TB group, which is consistent with our observation that palmitic acid was decreased in EBA samples collected from active TB cases. In another study, Weiner and coauthors reported a serum metabolic panel that could be used to distinguish between TB patients and healthy individuals^22^. That panel consisted of 20 metabolites, including phenylalanine which was slightly elevated in active TB patients, consistent with our findings. Using non-targeted metabolic analysis of plasma samples, Frediani and coauthors reported over 23,000 metabolites, of which 61 were found to be associated with pulmonary TB^21^; this included phosphatidylinositol, which was identified from one of 3 patients with multidrug-resistant tuberculosis (MDR-TB) and only one of 17 smear-negative adults, suggesting that this molecule was upregulated in TB subjects. In our study, two phosphatidylinositol species, PS 18:4 and PS 20:4, were prominent in active TB cases, with PS 24:4 showing the most separation power. Since this molecule is present commonly in both humans and *M. tuberculosis*, its source in active TB cases is unclear. On the other hand, it was noted that NAM, a common precursor of bacterial cell wall peptidoglycan, was much higher in GXP-positive cases.

Several limitations in data analysis should be acknowledged in our study. This study separated samples into sub-datasets based on study subjects’ clinical visits to reduce features. We were able to reduce the features from thousands to hundreds. However, the major drawback is that the 2-tailed *t*-test was conducted on a much smaller sample size that would identify less significant features. Therefore, we suggest that different feature reduction approaches should be conducted to optimize the results. We reported raw *p* values in the 2-tailed *t*-test. However, since the statistics were applied to the datasets containing more features than samples, the false discovery rate (FDR) needs to be considered, and the adjusted *p* values must be calculated. FDR adjustment should be a routine for metabolomic and lipidomic studies. However, they are not always reported by researchers. We reported adjusted *p* values using FDR thresholds between 0.05 and 0.2 (Supplemental Table 4). The metabolomic datasets showed that some features are no longer significant after an FDR of 0.05 was applied. The same trend is also observed when an FDR of 0.1 was applied. However, all features show significance when an FDR of 0.2 was applied. In the lipidomic datasets, all features still show significance after an FDR of 0.05 was applied, which agrees with the ROC curves in Fig. 8, from which we saw that lipids showed a stronger separation power than metabolites. Therefore, we suggest that the 22 molecules need to be further evaluated in another independent cohort study for diagnostic and therapeutic purposes.

## Conclusions

In this study, we demonstrated that rich NOCs can be extracted from human exhaled breath using an advanced analytical approach and the analysis could provide an attractive solution for human disease diagnosis. We advanced NOC analysis in human breath in several aspects. Using a comprehensive molecule extraction method, we have shown that hundreds of metabolites and lipids can be collected from human breath. Most importantly, the extraction method is rapid and has the potential to be used for high-volume sample processing in a clinical setting. By integrating MS-based non-targeted omics and sophisticated data analysis, we demonstrated that identified molecule signatures can differentiate active TB cases from healthy individuals based on the assigned importance of each molecule.

## Materials and methods

### Ethics statement and patient information

Written informed consent was acquired from all study participants and the study was approved by the University of Cape Town Human Research Ethics Committee (Reference number HREC680/2013). All experiments conducted in this study were performed in accordance with the relevant guidelines and regulations. In this study, we included 99 study participants. Since some study participants made multiple clinical visits and EBA samples were collected from each visit, the total sample size was 200. The study participants’ gender, age, HIV status, smoking status, and clinical visit times are included in Table 1 and Supplementary Table 1. Generally, exhaled breath aerosols and particles were collected in 10 mL of phosphate-buffered saline solution (PBS) using a wetted-wall cyclone air sampler (Coriolis µ Biological Air Sampler, Bertin Instruments, Montigny-le-Bretonneux, France) located in the respiratory aerosol sample chamber (RASC)^5,16,17^. For each study participant, collection was conducted for 60 min with an air flow rate of 240 L per minute. After collection, the liquid samples were processed with 0.2 µm polycarbonate filters (Sterlitech Corporation, WA, USA) to collect the bioaerosol pellets. The state of TB in the study participants was based on analysis of the bioaerosol pellet using GeneXpert PCR. The participants were identified as GeneXpert MTB/RIF (GXP-positive) or GXP-negative (control group). The supernatant from the filtration step was processed by dual phase extraction and analyzed by mass spectrometry (LC–MS/MS) as described in the following.

### Dual-phase extraction of metabolites and lipids in EBA samples

Chemicals and reagents were used in this study are either HPLC or MS-grade and commercially acquired from Fisher Scientific Chemical (Thermo Fisher Scientific, MA, USA). ( ±)-Methadone solution and insulin from bovine pancreas were purchased from MilliporeSigma (Burlington, MA). 1,2-Dilauroyl-sn-glycero-3-phosphorylcholine (DLPC) was purchased from Matreya, LLC (State College, PA). Filters and columns were purchased from Boca Scientific (Dedham, MA). 20 µm C18 beads were purchased from Hamilton (Reno, NV). C18 column packing methods were described previously^2^. For molecule extraction, each EBA sample (~ 10 mL) was loaded into a column and the liquid was pushed through using a syringe pump. The column was washed with 400 µL of 0.1% formic acid (FA) in water three times for cleaning and desalting. After desalting, two elution steps were performed. First, metabolites were eluted using 400 µL of 50% acetonitrile (ACN) in water. Next, lipids were eluted from the same column using 400 µL of 70% isopropyl alcohol (IPA). Both metabolite and lipid samples were lyophilized overnight. For metabolite analysis with MS, dried samples were resuspended in 0.05% trifluoroacetic acid (TFA) in water. For lipid analysis with MS, dried samples were suspended in 50% ACN. Samples containing MS-grade water were used as negative control samples. For quality control, a mixture of short peptides was used to monitor retention time, mass resolution, and accuracy (Thermo Fisher Scientific).

### Nano-liquid chromatography and MS

Metabolite and lipid samples were centrifuged at 10,000*g* for 10 min before being processed using an autosampler in an EASY-nLC 1000 system and characterized using a LTQ orbitrap system (Thermo Fisher Scientific). Chromatograms were generated using an Acclaim PepMap 100 C18 trap column (0.2 mm × 20 mm, 5 µL/min) and an analytical column (75 µm × 150 mm, 300 nL/min). The mobile phase used for metabolites was 80% ACN prepared in 0.1% FA in water from 5 to 70% in 60 min. The mobile phase used for lipids was 90% IPA and 10% ACN from 5 to 90% in 60 min. For accurate mass measurement, the resolution for MS was set to 30,000. Data collection was conducted using positive ion mode for both metabolites and lipids. High energy collision-induced disassociation was used for ion fragmentation with 35% total energy.

### Identification of metabolites and lipids using database matching

Mass spectra raw data files were converted to .abf format using an open-source software (reifycs.com/AbfConverter). Raw spectra were processed with MS-DIAL software for molecule identification following the standard operation procedures provided by the developers^23^. Additional methods for using MS-DIAL software for metabolomics and lipidomics were included in the supplementary information. The database for metabolites was extracted from MassBank, which includes 8068 records (as of August 9, 2021). The database for lipids was extracted from LipidBlast library, which contains 81 classes, 377,313 molecules, and 554,041 spectra (as of August 9, 2021)^24^.

### Data analysis and statistics

Metabolites and lipids identified using the software were extracted into Microsoft Excel. Since molecule identification generated features with different lengths, feature alignment and gap filling were conducted in MATLAB. Additional details were described in the supplementary information. An ion exclusion list was constructed using negative control samples. Total ion intensity was used for data normalization. For statistical analysis, ion intensity was log-transformed. For each patient visit, relative standard deviation (%) of each identified molecule was calculated and subsequently ranked in the GXP-negative and GXP-positive groups. Molecules with the lowest relative standard deviation values (30% cutoff) were processed with a *t*-test between GXP-negative and GXP-positive.

Statistically significant features calculated via a *t*-test from all 3 clinical visits were used for generating ROC curves. The area under the ROC curve (A.U.C) was calculated using the logistic regression model of identified molecules. In our previous study, a feature ranking algorithm, significance analysis of microarrays (SAM), was used to select the most powerful feature that separates the two groups^5^. In this study, SAM was applied to statistically significant features in all 3 clinical visits and the scores calculated from SAM analysis were used to determine the significance of each feature. RStudio was used to generate ROC curves and SAM and the methods are included in the supplementary information. To evaluate the data normality, Shapiro–Wilk test was conducted in the dataset of each visit and the results are included in the supplementary information.

## Supplementary Information

Supplementary Information 1.

Supplementary Information 2.

Supplementary Information 3.

Supplementary Information 4.

Supplementary Information 5.

## Data Availability

The data that support the findings of this study are available on request from the corresponding author. The datasets generated and/or analyzed during the current study are not publicly available due to their containing information that could compromise the privacy of research participants.
