# Supplementary material for: Non-volatile organic compounds in exhaled breath particles correspond to active tuberculosis
Source: Sci Rep. 2022 May 13;12:7919. doi: 10.1038/s41598-022-12018-6 (PMC9106714; doi:10.1038/s41598-022-12018-6)
Supplement: Supplementary file 1 — Supplementary Information 1. [file 41598_2022_12018_MOESM1_ESM.pdf]

Study ID	TB Status	Gender	HIV Status	Age	Smoking Information		Clinical Visit & Sample
	GXP				Status	Daily	
1	Negative	Female	Negative	22	Non-smoker	0	3
2	Negative	Female	Negative	35	Smoker	1	3
3	Negative	Female	Negative	21	Smoker	1	3
4	Negative	Female	Negative	18	Smoker	4	3
5	Negative	Female	Negative	34	Non-smoker	0	3
6	Negative	Female	Negative	19	Smoker	3	3
7	Negative	Female	Negative	25	Non-smoker	0	3
8	Negative	Female	Negative	21	Smoker	4	3
9	Negative	Female	Negative	31	Smoker	2	3
10	Negative	Female	Negative	41	Non-smoker	0	2
11	Negative	Female	Negative	29	Non-smoker	0	3
12	Negative	Male	Negative	24	Smoker	2	3
13	Negative	Male	Negative	30	Smoker	3	3
14	Negative	Male	Negative	59	Smoker	4	3
15	Negative	Female	Negative	32	Non-smoker	0	3
16	Negative	Female	Negative	21	Non-smoker	0	3
17	Negative	Female	Negative	18	Non-smoker	0	3
18	Negative	Male	Negative	33	Smoker	10	3
19	Negative	Male	Negative	24	Smoker	4	2
20	Negative	Male	Negative	21	Smoker	6	3
21	Negative	Male	Negative	53	Non-smoker	0	3
22	Negative	Female	Negative	28	Non-smoker	0	3
23	Negative	Female	Negative	31	Non-smoker	0	3
24	Negative	Male	Negative	20	Non-smoker	0	3
25	Negative	Male	Negative	35	Smoker	6	3
26	Negative	Female	Negative	19	Smoker	4	3

27	Negative	Female	Negative	35	Non-smoker	0	3
28	Negative	Female	Negative	19	Non-smoker	0	3
29	Negative	Female	Negative	52	Non-smoker	0	3
30	Negative	Male	Negative	40	Smoker	4	3
31	Negative	Female	Negative	19	Non-smoker	0	3
32	Negative	Female	Negative	31	Non-smoker	0	3
33	Negative	Male	Negative	26	Smoker	3	3
34	Negative	Male	Negative	20	Smoker	5	2
35	Negative	Male	Negative	26	Smoker	5	3
36	Negative	Female	Negative	39	Smoker	5	3
37	Negative	Male	Negative	25	Non-smoker	0	4
38	Negative	Female	Negative	42	Non-smoker	0	3
39	Negative	Female	Negative	21	Non-smoker	0	3
40	Negative	Male	Negative	20	Smoker	2	3
41	Negative	Female	Negative	18	Smoker	5	2
42	Negative	Male	Negative	19	Smoker	5	3
43	Negative	Male	Negative	20	Smoker	5	3
44	Negative	Female	Negative	23	Non-smoker	0	2
45	Negative	Male	Negative	29	Smoker	10	3
46	Negative	Male	Negative	51	Smoker	3	3
47	Negative	Female	Negative	26	Smoker	2	3
48	Negative	Female	Negative	28	Non-smoker	0	3
49	Negative	Female	Negative	21	Non-smoker	0	3
50	Negative	Female	Negative	23	Smoker	6	3
52	Negative	Female	Negative	18	Non-smoker	0	3
53	Negative	Female	Negative	26	Non-smoker	0	1
54	Negative	Female	Negative	31	Smoker	6	3
55	Negative	Female	Negative	24	Smoker	3	3

56	Negative	Male	Negative	34	Smoker	15	2
57	Negative	Female	Negative	26	Non-smoker	0	2
59	Negative	Male	Negative	54	Smoker	3	2
60	Negative	Male	Negative	32	Smoker	15	1
61	Negative	Male	Negative	39	Smoker	20	2
62	Negative	Female	Negative	23	Non-smoker	0	2
63	Negative	Female	Negative	19	Smoker	3	2
64	Negative	Female	Negative	40	Smoker	4	2
66	Negative	Female	Negative	19	Non-smoker	0	3
67	Negative	Female	Negative	23	Non-smoker	0	3
68	Negative	Female	Negative	24	Non-smoker	0	3
69	Negative	Female	Negative	35	Non-smoker	0	1
70	Negative	Female	Negative	58	Non-smoker	0	1
71	Negative	Female	Negative	24	Non-smoker	0	2
72	Negative	Female	Negative	21	Non-smoker	0	2
73	Negative	Male	Negative	32	Smoker	3	2
74	Negative	Female	Negative	30	Non-smoker	0	2
75	Negative	Male	Negative	28	Non-smoker	0	2
80	Negative	Male	Negative	26	Smoker	3	3
51	Positive	Male	Negative	36	Smoker	10	1
58	Positive	Female	Negative	27	Non-smoker	0	1
65	Positive	Female	Negative	35	Non-smoker	0	1
76	Positive	Male	Negative	28	Non-smoker	0	4
77	Positive	Male	Negative	43	Non-smoker	0	2
78	Positive	Male	Negative	28	Smoker	8	1
79	Positive	Female	Negative	19	Smoker	3	1
81	Positive	Male	Negative	49	Smoker	4	3
82	Positive	Female	Negative	51	Non-smoker	0	3

83	Positive	Male	Negative	26	Smoker	7	4
84	Positive	Male	Negative	48	Smoker	6	4
85	Positive	Male	Negative	45	Smoker	10	4
86	Positive	Male	Negative	49	Smoker	3	4
87	Positive	Female	Negative	38	Smoker	3	4
88	Positive	Female	Negative	19	Non-smoker	0	4
89	Positive	Female	Negative	21	Non-smoker	0	4
90	Positive	Male	Negative	19	Smoker	20	2
91	Positive	Male	Negative	28	Non-smoker	0	4
92	Positive	Male	Negative	28	Non-smoker	0	4
93	Positive	Male	Negative	38	Smoker	4	4
94	Positive	Male	Negative	30	Smoker	5	3
95	Positive	Male	Negative	21	Smoker	3	3
96	Positive	Male	Negative	29	Smoker	3	1
97	Positive	Male	Negative	31	Smoker	20	1
98	Positive	Male	Negative	54	Smoker	5	2
99	Positive	Male	Negative	39	Smoker	20	1
