# Supplementary material for: Non-volatile organic compounds in exhaled breath particles correspond to active tuberculosis
Source: Sci Rep. 2022 May 13;12:7919. doi: 10.1038/s41598-022-12018-6 (PMC9106714; doi:10.1038/s41598-022-12018-6)
Supplement: Supplementary file 2 — Supplementary Information 2. [file 41598_2022_12018_MOESM2_ESM.pdf]

| 1st visit | | | 2nd visit | | | 3rd visit | | |
|---|---|---|---|---|---|---|---|---|
| Features | GXP positive/negative [Log2] | p value -[Log10] | Features | GXP positive/negative [Log2] | p value -[Log10] | Features | GXP positive/negative [Log2] | p value -[Log10] |
| | Metabolites | | | | | | | |
| Uridine | 0.89 | 1.35 | NAM | 0.97 | 1.54 | NAM | 0.35 | 1.33 |
| NAM | 0.89 | 1.82 | Uridine | 0.97 | 1.40 | Uridine | 0.34 | 1.36 |
| Kynurenine | 0.51 | 2.05 | Kynurenine | 0.93 | 1.64 | Kynurenine | 0.30 | 1.36 |
| GLN | 0.45 | 1.54 | iPEN | 0.93 | 1.69 | GLN | 0.27 | 1.87 |
| iPEN | 0.42 | 1.64 | GLN | 0.90 | 1.87 | iPEN | 0.25 | 1.68 |
| Guanosine | 0.37 | 1.54 | PHE | 0.90 | 1.70 | Guanosine | 0.25 | 1.59 |
| PHE | 0.37 | 1.46 | Proline | 0.75 | 1.64 | PHE | 0.24 | 1.48 |
| Proline | 0.34 | 2.05 | Guanosine | 0.73 | 1.87 | Proline | 0.23 | 2.61 |
| PA | -0.23 | 1.44 | G6P | -0.21 | 1.51 | HA | -0.60 | 1.78 |
| BA | -0.23 | 1.59 | AMP | -0.47 | 1.54 | G6P | -0.61 | 1.50 |
| G6P | -0.59 | 1.47 | PA | -0.48 | 1.64 | PA | -0.63 | 1.46 |
| HA | -0.74 | 1.40 | BA | -0.49 | 1.60 | AMP | -0.63 | 2.80 |
| AMP | -0.92 | 1.39 | HA | -0.50 | 1.70 | BA | -0.73 | 2.06 |
| | Lipids | | | | | | | |
| PS 24:4 | 1.58 | 3.71 | PI 18:4 | 1.62 | 2.41 | Cer 8:0 | 0.53 | 3.53 |
| Cer 8:0 | 1.07 | 3.25 | PS 24:4 | 1.48 | 3.71 | PI 18:4 | 0.49 | 2.42 |
| PI 18:4 | 0.94 | 4.64 | Cer 8:0 | 1.21 | 2.79 | PS 24:4 | 0.25 | 2.33 |
| DG O-8:0 | 0.93 | 2.60 | PI 20:4 | 1.07 | 2.51 | PI 20:4 | 0.25 | 3.20 |
| PI 20:4 | 0.91 | 4.18 | PC 64:5 | 0.94 | 2.32 | DG O-8:0 | 0.25 | 2.97 |
| PC 64:5 | 0.70 | 2.41 | DG O-8:0 | 0.88 | 2.97 | PC 64:5 | 0.24 | 4.13 |
| Cer 18:0 | 0.51 | 2.97 | Cer 18:0 | 0.79 | 2.60 | Cer 18:0 | 0.24 | 3.25 |
| PC 56:7 | -0.52 | 1.86 | TG 9:0 | -0.40 | 1.39 | PC 56:7 | -0.39 | 1.67 |
| TG 9:0 | -0.93 | 1.35 | PC 56:7 | -1.22 | 1.36 | TG 9:0 | -0.41 | 1.39 |
