# Supplementary material for: Non-volatile organic compounds in exhaled breath particles correspond to active tuberculosis
Source: Sci Rep. 2022 May 13;12:7919. doi: 10.1038/s41598-022-12018-6 (PMC9106714; doi:10.1038/s41598-022-12018-6)
Supplement: Supplementary file 3 — Supplementary Information 3. [file 41598_2022_12018_MOESM3_ESM.pdf]

Metabolites	Group	Average	Standard deviation	Shapiro-Wilk *p* value
G6P	GXP-pos	6.30	1.10	0.756
	GXP-neg	4.93	1.18	0.213
AMP	GXP-pos	6.12	1.20	0.563
	GXP-neg	4.98	1.12	0.127
BA	GXP-pos	5.84	1.31	0.853
	GXP-neg	4.76	1.43	0.214
Uridine	GXP-pos	5.03	1.38	0.723
	GXP-neg	7.10	0.87	0.632
Proline	GXP-pos	5.63	1.09	0.822
	GXP-neg	6.74	1.11	0.423
Guanosine	GXP-pos	5.70	1.12	0.473
	GXP-neg	6.40	1.11	0.322
Kynurenine	GXP-pos	4.80	1.38	0.743
	GXP-neg	7.19	0.82	0.239
PA	GXP-pos	6.48	1.15	0.42
	GXP-neg	4.62	1.00	0.239
NAM	GXP-pos	4.53	1.07	0.328
	GXP-neg	6.55	1.10	0.145
PHE	GXP-pos	5.88	1.46	0.637
	GXP-neg	6.95	0.84	0.48
HA	GXP-pos	6.14	1.40	0.392
	GXP-neg	4.81	1.01	0.282
GLN	GXP-pos	5.49	1.13	0.333
	GXP-neg	6.81	1.05	0.185
iPEN	GXP-pos	5.64	1.11	0.423
	GXP-neg	6.60	1.07	0.299

Lipids	Group	Average	Standard deviation	Shapiro-Wilk *p* value
PS 24:4	GXP-pos	3.47	0.83	0.741
	GXP-neg	7.43	0.81	0.254
Cer 8:0	GXP-pos	5.02	1.11	0.523
	GXP-neg	8.00	1.12	0.284
PI 20:4	GXP-pos	4.52	0.81	0.627
	GXP-neg	7.21	0.76	0.471
DG O-8:0	GXP-pos	4.04	1.11	0.544
	GXP-neg	7.24	1.41	0.289
PC 56:7	GXP-pos	7.06	0.85	0.478
	GXP-neg	6.48	1.36	0.12
Cer 18:0	GXP-pos	5.44	1.42	0.712
	GXP-neg	6.38	1.10	0.421
TG 9:0	GXP-pos	6.64	1.11	0.387
	GXP-neg	5.74	1.15	0.145
PI 18:4	GXP-pos	5.12	0..81	0.698
	GXP-neg	7.56	0.75	0.423
PC 64:5	GXP-pos	6.44	1.70	0.711
	GXP-neg	6.93	1.46	0.192
