# Supplementary material for: Non-volatile organic compounds in exhaled breath particles correspond to active tuberculosis
Source: Sci Rep. 2022 May 13;12:7919. doi: 10.1038/s41598-022-12018-6 (PMC9106714; doi:10.1038/s41598-022-12018-6)
Supplement: Supplementary file 4 — Supplementary Information 4. [file 41598_2022_12018_MOESM4_ESM.pdf]

Benjamini-Hochberg Method													
		FDR (0.05)				FDR (0.1)				FDR (0.2)			
	Rank	Raw P value	Critical Value	Adjusted P value	Significance with FDR	Raw P value	Critical Value	Adjusted P value	Significance with FDR	Raw P value	Critical Value	Adjusted P value	Significance with FDR
1st visit	Metabolites												
	3	0.0089	0.0038	0.1157	No	0.0089	0.0077	0.1157	No	0.0089	0.0154	0.1157	Yes
	4	0.0089	0.0077	0.0579	No	0.0089	0.0154	0.0579	Yes	0.0089	0.0308	0.0579	Yes
	8	0.0150	0.0115	0.0649	No	0.0150	0.0231	0.0649	Yes	0.0150	0.0462	0.0649	Yes
	14	0.0229	0.0154	0.0744	No	0.0229	0.0308	0.0744	Yes	0.0229	0.0615	0.0744	Yes
	16	0.0258	0.0192	0.0670	No	0.0258	0.0385	0.0670	Yes	0.0258	0.0769	0.0670	Yes
	21	0.0288	0.0231	0.0625	No	0.0288	0.0462	0.0625	Yes	0.0288	0.0923	0.0625	Yes
	22	0.0290	0.0269	0.0538	No	0.0290	0.0538	0.0538	Yes	0.0290	0.1077	0.0538	Yes
	26	0.0342	0.0308	0.0556	No	0.0342	0.0615	0.0556	Yes	0.0342	0.1231	0.0556	Yes
	27	0.0350	0.0346	0.0506	No	0.0350	0.0692	0.0506	Yes	0.0350	0.1385	0.0506	Yes
	28	0.0367	0.0385	0.0477	Yes	0.0367	0.0769	0.0477	Yes	0.0367	0.1538	0.0477	Yes
	30	0.0394	0.0423	0.0466	Yes	0.0394	0.0846	0.0466	Yes	0.0394	0.1692	0.0466	Yes
	31	0.0408	0.0462	0.0442	Yes	0.0408	0.0923	0.0442	Yes	0.0408	0.1846	0.0442	Yes
	35	0.0451	0.0500	0.0451	Yes	0.0451	0.1000	0.0451	Yes	0.0451	0.2000	0.0451	Yes
	Lipids												
	2	2.3E-05	5.6E-03	2.1E-04	Yes	2.3E-05	1.1E-02	2.1E-04	Yes	2.3E-05	2.2E-02	2.1E-04	Yes
	3	6.7E-05	1.1E-02	3.0E-04	Yes	6.7E-05	2.2E-02	3.0E-04	Yes	6.7E-05	4.4E-02	3.0E-04	Yes
	9	1.9E-04	1.7E-02	5.8E-04	Yes	1.9E-04	3.3E-02	5.8E-04	Yes	1.9E-04	6.7E-02	5.8E-04	Yes
	13	5.6E-04	2.2E-02	1.3E-03	Yes	5.6E-04	4.4E-02	1.3E-03	Yes	5.6E-04	8.9E-02	1.3E-03	Yes
	16	1.1E-03	2.8E-02	1.9E-03	Yes	1.1E-03	5.6E-02	1.9E-03	Yes	1.1E-03	1.1E-01	1.9E-03	Yes
	21	0.0025	0.0333	0.0038	Yes	0.0025	0.0667	0.0038	Yes	0.0025	0.1333	0.0038	Yes
	26	0.0039	0.0389	0.0050	Yes	0.0039	0.0778	0.0050	Yes	0.0039	0.1556	0.0050	Yes
	37	0.0139	0.0444	0.0157	Yes	0.0139	0.0889	0.0157	Yes	0.0139	0.1778	0.0157	Yes
	48	0.0451	0.0500	0.0451	Yes	0.0451	0.1000	0.0451	Yes	0.0451	0.2000	0.0451	Yes
	Metabolites												
	18	0.0135	0.0038	0.1759	No	0.0135	0.0077	0.1759	No	0.0135	0.0154	0.1759	Yes
	19	0.0135	0.0077	0.0879	No	0.0135	0.0154	0.0879	Yes	0.0135	0.0308	0.0879	Yes
	28	0.0202	0.0115	0.0873	No	0.0202	0.0231	0.0873	Yes	0.0202	0.0462	0.0873	Yes
	29	0.0202	0.0154	0.0655	No	0.0202	0.0308	0.0655	Yes	0.0202	0.0615	0.0655	Yes

**2nd visit**	30	0.0203	0.0192	0.0527	No	0.0203	0.0385	0.0527	Yes	0.0203	0.0769	0.0527	Yes
	34	0.0231	0.0231	0.0501	No	0.0231	0.0462	0.0501	Yes	0.0231	0.0923	0.0501	Yes
	35	0.0231	0.0269	0.0429	Yes	0.0231	0.0538	0.0429	Yes	0.0231	0.1077	0.0429	Yes
	36	0.0231	0.0308	0.0376	Yes	0.0231	0.0615	0.0376	Yes	0.0231	0.1231	0.0376	Yes
	39	0.0250	0.0346	0.0361	Yes	0.0250	0.0692	0.0361	Yes	0.0250	0.1385	0.0361	Yes
	46	0.0287	0.0385	0.0373	Yes	0.0287	0.0769	0.0373	Yes	0.0287	0.1538	0.0373	Yes
	47	0.0287	0.0423	0.0339	Yes	0.0287	0.0846	0.0339	Yes	0.0287	0.1692	0.0339	Yes
	54	0.0311	0.0462	0.0337	Yes	0.0311	0.0923	0.0337	Yes	0.0311	0.1846	0.0337	Yes
	63	0.0397	0.0500	0.0397	Yes	0.0397	0.1000	0.0397	Yes	0.0397	0.2000	0.0397	Yes
	**Lipids**												
	4	1.9E-04	5.6E-03	1.7E-03	Yes	1.9E-04	1.1E-02	1.7E-03	Yes	1.9E-04	2.2E-02	1.7E-03	Yes
	7	1.1E-03	1.1E-02	4.8E-03	Yes	1.1E-03	2.2E-02	4.8E-03	Yes	1.1E-03	4.4E-02	4.8E-03	Yes
	13	1.6E-03	1.7E-02	4.9E-03	Yes	1.6E-03	3.3E-02	4.9E-03	Yes	1.6E-03	6.7E-02	4.9E-03	Yes
	20	2.5E-03	2.2E-02	5.7E-03	Yes	2.5E-03	4.4E-02	5.7E-03	Yes	2.5E-03	8.9E-02	5.7E-03	Yes
	23	3.1E-03	2.8E-02	5.6E-03	Yes	3.1E-03	5.6E-02	5.6E-03	Yes	3.1E-03	1.1E-01	5.6E-03	Yes
	28	0.0039	0.0333	0.0058	Yes	0.0039	0.0667	0.0058	Yes	0.0039	0.1333	0.0058	Yes
	31	0.0048	0.0389	0.0061	Yes	0.0048	0.0778	0.0061	Yes	0.0048	0.1556	0.0061	Yes
	92	0.0405	0.0444	0.0455	Yes	0.0405	0.0889	0.0455	Yes	0.0405	0.1778	0.0455	Yes
	96	0.0432	0.0500	0.0432	Yes	0.0432	0.1000	0.0432	Yes	0.0432	0.2000	0.0432	Yes
**3rd visit**	**Metabolites**												
	1	0.0016	0.0038	0.0204	Yes	0.0016	0.0077	0.0204	Yes	0.0016	0.0154	0.0204	Yes
	2	0.0025	0.0077	0.0160	Yes	0.0025	0.0154	0.0160	Yes	0.0025	0.0308	0.0160	Yes
	6	0.0088	0.0115	0.0381	Yes	0.0088	0.0231	0.0381	Yes	0.0088	0.0462	0.0381	Yes
	23	0.0135	0.0154	0.0439	Yes	0.0135	0.0308	0.0439	Yes	0.0135	0.0615	0.0439	Yes
	27	0.0168	0.0192	0.0436	Yes	0.0168	0.0385	0.0436	Yes	0.0168	0.0769	0.0436	Yes
	32	0.0208	0.0231	0.0450	Yes	0.0208	0.0462	0.0450	Yes	0.0208	0.0923	0.0450	Yes
	35	0.0258	0.0269	0.0479	Yes	0.0258	0.0538	0.0479	Yes	0.0258	0.1077	0.0479	Yes
	40	0.0320	0.0308	0.0519	No	0.0320	0.0615	0.0519	Yes	0.0320	0.1231	0.0519	Yes
	43	0.0334	0.0346	0.0482	Yes	0.0334	0.0692	0.0482	Yes	0.0334	0.1385	0.0482	Yes
	47	0.0348	0.0385	0.0453	Yes	0.0348	0.0769	0.0453	Yes	0.0348	0.1538	0.0453	Yes
	59	0.0432	0.0423	0.0511	No	0.0432	0.0846	0.0511	Yes	0.0432	0.1692	0.0511	Yes
	61	0.0441	0.0462	0.0478	Yes	0.0441	0.0923	0.0478	Yes	0.0441	0.1846	0.0478	Yes
	65	0.0467	0.0500	0.0467	Yes	0.0467	0.1000	0.0467	Yes	0.0467	0.2000	0.0467	Yes
	**Lipids**												

	1	7.5E-05	5.6E-03	6.7E-04	Yes	7.5E-05	1.1E-02	6.7E-04	Yes	7.5E-05	2.2E-02	6.7E-04	Yes
	5	2.9E-04	1.1E-02	1.3E-03	Yes	2.9E-04	2.2E-02	1.3E-03	Yes	2.9E-04	4.4E-02	1.3E-03	Yes
	7	5.6E-04	1.7E-02	1.7E-03	Yes	5.6E-04	3.3E-02	1.7E-03	Yes	5.6E-04	6.7E-02	1.7E-03	Yes
	8	6.3E-04	2.2E-02	1.4E-03	Yes	6.3E-04	4.4E-02	1.4E-03	Yes	6.3E-04	8.9E-02	1.4E-03	Yes
	9	1.1E-03	2.8E-02	1.9E-03	Yes	1.1E-03	5.6E-02	1.9E-03	Yes	1.1E-03	1.1E-01	1.9E-03	Yes
	18	0.0038	0.0333	0.0058	Yes	0.0038	0.0667	0.0058	Yes	0.0038	0.1333	0.0058	Yes
	20	0.0047	0.0389	0.0060	Yes	0.0047	0.0778	0.0060	Yes	0.0047	0.1556	0.0060	Yes
	37	0.0213	0.0444	0.0239	Yes	0.0213	0.0889	0.0239	Yes	0.0213	0.1778	0.0239	Yes
	52	0.0403	0.0500	0.0403	Yes	0.0403	0.1000	0.0403	Yes	0.0403	0.2000	0.0403	Yes
