# Supplementary material for: Non-volatile organic compounds in exhaled breath particles correspond to active tuberculosis
Source: Sci Rep. 2022 May 13;12:7919. doi: 10.1038/s41598-022-12018-6 (PMC9106714; doi:10.1038/s41598-022-12018-6)
Supplement: Supplementary file 5 — Supplementary Information 5. [file 41598_2022_12018_MOESM5_ESM.pdf]

Supporting Information

Non-volatile Organic Compounds in Exhaled Breath Particles Correspond to Active Tuberculosis

Dapeng Chen[1]*, Noella A. Bryden[1], Wayne A. Bryden[1], Michael McLoughlin[1], Dexter Smith[1], Alese P. Devin[1], Emily R. Caton[1], Caroline R. Haddaway[1], Michele Tameris[2], Thomas J. Scriba[2], Mark Hatherill[2], Sophia Gessner[3], Digby F. Warner[3], Robin Wood[4]
[1] Zeteo Tech, Inc., Sykesville, Maryland, United States of America
[2] South African Tuberculosis Vaccine Initiative, Institute of Infectious Disease and Molecular Medicine and Division of Immunology, Department of Pathology, University of Cape Town, South Africa
[3] SAMRC/NHLS/UCT Molecular Mycobacteriology Research Unit, Institute of Infectious Disease and Molecular Medicine and Division of Medical Microbiology, Department of Pathology, University of Cape Town, South Africa
[4] Desmond Tutu HIV Centre, Institute of Infectious Diseases and Molecular Medicine, University of Cape Town, Cape Town, South Africa
*To whom correspondence should be addressed: dapeng.chen@zeteotech.com

Supplementary Methods

MS-DIAL for metabolomics and lipidomics

MS-DIAL can be downloaded from the software website (http://prime.psc.riken.jp/compms/msdial/main.html). To start the projects for our analysis, LC/MS option was selected. Since the MS/MS was collected in the ion trap, profile was selected for MS1 and centroid option was selected for MS/MS. For molecule identification using database searching, adduct ion information was required. For this purpose, [M+H]+ and [M+Na]+ were selected. MS1 tolerance was defined as 0.01 Da. MS2 tolerance was defined as 0.025 Da. Retention time begin was defined as 5 min and end was defined as 75 min. The MS1 mass range begin was defined as 100 Da and the end defined as 2000 Da. For isotope recognition, the maximum charged number was defined as 2. For the peak detection, the minimum peak height was defined as 5000 amplitude and the mass slice width was defined as 0.1 Da. The advance option was set to default. In the MS2Dec option, the sigma window value was defined as 0.1 and MS/MS abundance cut off was defined as 5. Other option parameter selection can be requested by contacting with the corresponding author.

ROC curve construction and SAM analysis using RStudio

An open-source package for R to analyze and compare ROC curves was published (Ref 1) and the package can be installed in RStudio:

```
# install.packages("pROC")

# library(pROC)
```

The function of roc and plot.roc used in our study was shown below:

```
--------------------
> roc
function (...)
{
   UseMethod("roc")
}
<bytecode: 0x000002d29ebbc1b0>
<environment: namespace:pROC>
--------------------
```

```
# plot.roc
function (x, ...)
{
    UseMethod("plot.roc")
}
<bytecode: 0x000002d29ec3acc0>
<environment: namespace:pROC>
---------------------
```

SAM analysis was published (Ref 2) and the package can be installed in RStudio:

```
# library(devtools)

# install_github("cran/samr")

# install.packages("samr")

# library(samr)
```

SAM

```
function (x, y = NULL, censoring.status = NULL, resp.type = c("Quantitative",
    "Two class unpaired", "Survival", "Multiclass",
    "One class", "Two class paired", "Two class unpaired timecourse",
    "One class timecourse", "Two class paired timecourse",
    "Pattern discovery"), geneid = NULL, genenames = NULL,
    s0 = NULL, s0.perc = NULL, nperms = 100, center.arrays = FALSE,
    testStatistic = c("standard", "wilcoxon"), time.summary.type = c("slope",
        "signed.area"), regression.method = c("standard",
        "ranks"), return.x = TRUE, knn.neighbors = 10,
    random.seed = NULL, logged2 = FALSE, fdr.output = 0.2, eigengene.number = 1)
{
    this.call <- match.call()
    xl.mode = "regular"
    xl.time = NULL
    xl.prevfit = NULL
    if (fdr.output < 0 | fdr.output > 1) {
        stop("Error: fdr.output must be between 0 and 1")
    }
    if (is.null(geneid)) {
        geneid = as.character(1:nrow(x))
    }
    if (is.null(genenames)) {
        genenames = paste("g", as.character(1:nrow(x)),
            sep = "")
    }
    data = list(x = x, y = y, censoring.status = censoring.status,
        geneid = geneid, genenames = genenames, logged2 = logged2,
        eigengene.number = eigengene.number)
    samr.obj = samr(data, resp.type = resp.type, assay.type = "array",
        s0 = s0, s0.perc = s0.perc, nperms = nperms, center.arrays = center.arrays,
        testStatistic = testStatistic, time.summary.type = time.summary.type,
```

```
        regression.method = regression.method, return.x = return.x,
        knn.neighbors = knn.neighbors, random.seed = random.seed)
    delta.table <- samr.compute.delta.table(samr.obj)
    siggenes.table <- del <- NULL
    delta.table <- delta.table[delta.table[, "# called"] >
        0, , drop = FALSE]
    if (nrow(delta.table) > 0) {
        oo <- which(delta.table[, "median FDR"] >= fdr.output)
        if (length(oo) > 0) {
            oo <- oo[length(oo)]
        }
        else {
            oo <- 1
        }
        delta.table <- delta.table[oo:nrow(delta.table), , drop = FALSE]
        del <- delta.table[1, "delta"]
        siggenes.table <- samr.compute.siggenes.table(samr.obj,
            del, data, delta.table)
        rang = 4:8
        if (resp.type == "Multiclass") {
            nclass = length(table(y))
            rang = 3:(ncol(siggenes.table$genes.up))
        }
        if (resp.type == "Quantitative" | resp.type ==
            "Pattern discovery" | resp.type == "Survival") {
            rang = 4:7
        }
        siggenes.table$genes.up[, rang] = round(as.numeric(siggenes.table$genes.up[,
            rang]), 3)
        siggenes.table$genes.lo[, rang] = round(as.numeric(siggenes.table$genes.lo[,
            rang]), 3)
        siggenes.table$genes.up = siggenes.table$genes.up[, -1]
        siggenes.table$genes.lo = siggenes.table$genes.lo[, -1]
    }
    out = list(samr.obj = samr.obj, del = del, delta.table = delta.table,
        siggenes.table = siggenes.table)
    out$call = this.call
    class(out) = "SAMoutput"
    return(out)
}
<bytecode: 0x000002d2a770f2a8>
<environment: namespace:samr>
```

Shapiro-Wilk normality test

The Shapiro-Wilk test for multivariate normality was conducted in Rstudio and the method was based on the published work (3-6).

The package can be installed:

```
# library(mvnormtest)
```

The function # mshapiro.test was described below:

```
function (U)
{
   if (!is.matrix(U))
      stop("U[] is not a matrix with number of columns (sample size) between 3 and 5000")
   n <- ncol(U)
   if (n < 3 || n > 5000)
      stop("sample size must be between 3 and 5000")
   rng <- range(U)
   rng <- rng[2] - rng[1]
   if (rng == 0)
      stop("all `U[]' are identical")
   Us <- apply(U, 1, mean)
   R <- U - Us
   M.1 <- solve(R %*% t(R), tol = 1e-18)
   Rmax <- diag(t(R) %*% M.1 %*% R)
   C <- M.1 %*% R[, which.max(Rmax)]
   Z <- t(C) %*% U
   return(shapiro.test(Z))
}
<bytecode: 0x000002d2a79313d0>
<environment: namespace:mvnormtest>
```

Supplementary Figure:

Figure S1. Shapiro-Wilk test for multivariate normality in the dataset of each visit.

Supplementary Tables:

Table S1. Additional study participant information.
Table S2: Volcano plot results of the 22 molecules identified in all 3 visits.
Table S3: Average, standard deviation, and Shapiro-Wilk analysis of the 22 molecules identified in all 3 visits.
Table S4: Adjusted *p* values using Benjamini-Hochberg method of the 22 molecules identified in all 3 visits.

A

	Molecule Type	Clinical Visit	Sample Size	*p* value
GXP positive	Metabolites	1st	20	0.756
		2nd	11	0.2834
		3rd	11	0.784
	Lipids	1st	20	0.9699
		2nd	11	0.5485
		3rd	11	0.4718
GXP negative	Metabolites	1st	26	0.1844
		2nd	63	0.27768
		3rd	53	0.2306
	Lipids	1st	26	0.3699
		2nd	63	0.4123
		3rd	53	0.8922

B

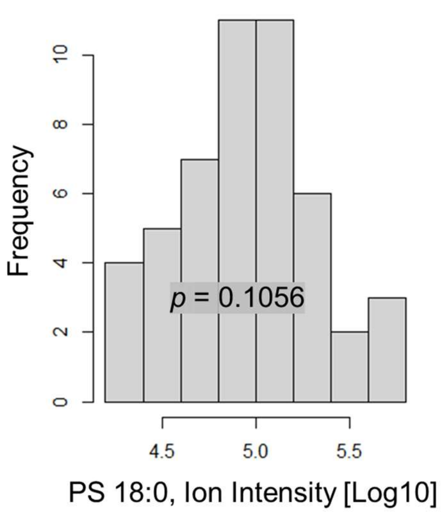

Fig. S1. Shapiro-Wilk test for multivariate normality in the dataset of each visit (A) and a lipid molecule, PS 18:0, in the GXP negative 3rd visit dataset (B). The p-value of each test was larger than .05, which showed that the sample data came from a population that was normally distributed. A molecule in B was used as a representative plot to show the data normality.

Ref 1. Robin, X., Turck, N., Hainard, A., Tiberti, N., Lisacek, F., Sanchez, J.C. and Müller, M., 2011. pROC: an open-source package for R and S+ to analyze and compare ROC curves. BMC bioinformatics, 12(1), pp.1-8.
Ref 2. Tusher, V.G., Tibshirani, R. and Chu, G., 2001. Significance analysis of microarrays applied to the ionizing radiation response. Proceedings of the National Academy of Sciences, 98(9), pp.5116-5121.
Ref 3. Czeslaw Domanski (1998) Wlasnosci testu wielowymiarowej normalnosci Shapiro-Wilka i jego zastosowanie. Cracow University of Economics Rector's Lectures, No. 37.
Ref 4. Patrick Royston (1982) An Extension of Shapiro and Wilk's W Test for Normality to Large Samples. Applied Statistics, 31, 115–124.
Ref 5. Patrick Royston (1982) Algorithm AS 181: The W Test for Normality. Applied Statistics, 31, 176–180.
Ref 6. Patrick Royston (1995) A Remark on Algorithm AS 181: The W Test for Normality. Applied Statistics, 44, 547–551.
